# *N*,*N*-Dimethyl­ethane-1,2-diaminium bis­(3-hy­droxy­benzoate)

**DOI:** 10.1107/S1600536811040578

**Published:** 2011-10-08

**Authors:** Anindita Sarkar, Ignacy Cukrowski

**Affiliations:** a Department of Chemistry, University of Pretoria, Lynnwood Road, Pretoria 0002, South Africa

## Abstract

In the title compound, C_4_H_14_N_2_^2+^·2C_7_H_5_O_3_^−^, both the *N*,*N*-dimethyl­ethylenediamine N atoms are protonated and two 3-hy­droxy­benzoate anions act as counter-ions. In the crystal, anions and cations are linked by a network of N—H⋯O and O—H⋯O hydrogen bonds.

## Related literature

For bond lengths in fully protonated polyamines, see: Bujak & Angel (2006 ▶); Bujak & Zaleski (2002 ▶); Doran *et al.* (2003 ▶); Thorn *et al.* (2005 ▶); Zhang *et al.* (2007 ▶). 
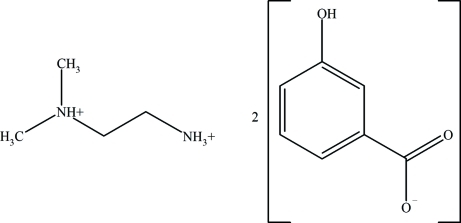


## Experimental

### 

#### Crystal data

C_4_H_14_N_2_^2+^·2C_7_H_5_O_3_^−^
*M* *_r_* = 364.39
Monoclinic, 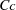

*a* = 14.5439 (3) Å
*b* = 17.5881 (4) Å
*c* = 7.7104 (2) Å
β = 114.777 (1)°
*V* = 1790.76 (7) Å^3^
*Z* = 4
Mo *K*α radiation
μ = 0.10 mm^−1^
*T* = 293 K
0.49 × 0.12 × 0.03 mm

#### Data collection

Bruker APEXII CCD diffractometer
Absorption correction: integration (*XPREP*; Bruker, 2001 ▶) *T*_min_ = 0.952, *T*_max_ = 0.997
14114 measured reflections
4292 independent reflections
3635 reflections with *I* > 2σ(*I*)
*R*_int_ = 0.048

#### Refinement

*R*[*F* ^2^ > 2σ(*F* ^2^)] = 0.036
*wR*(*F* ^2^) = 0.077
*S* = 0.96
4292 reflections
240 parameters
2 restraints
H-atom parameters constrained
Δρ_max_ = 0.18 e Å^−3^
Δρ_min_ = −0.20 e Å^−3^
Absolute structure: Flack (1983 ▶), 2119 Friedel pairs
Flack parameter: 0.00 (7)

### 

Data collection: *APEX2* (Bruker, 2005 ▶); cell refinement: *SAINT* (Bruker, 2005 ▶); data reduction: *SAINT*; program(s) used to solve structure: *SHELXTL* (Sheldrick, 2008 ▶); program(s) used to refine structure: *SHELXTL* and *SHELXL97* (Sheldrick, 2008 ▶); molecular graphics: *ORTEP-3 for Windows* (Farrugia, 1997 ▶) and *Mercury* (Macrae *et al.*, 2006 ▶); software used to prepare material for publication: *SHELXL97* and *PLATON* (Spek, 2009 ▶).

## Supplementary Material

Crystal structure: contains datablock(s) I, global. DOI: 10.1107/S1600536811040578/fj2425sup1.cif

Structure factors: contains datablock(s) I. DOI: 10.1107/S1600536811040578/fj2425Isup2.hkl

Supplementary material file. DOI: 10.1107/S1600536811040578/fj2425Isup3.cml

Additional supplementary materials:  crystallographic information; 3D view; checkCIF report

## Figures and Tables

**Table 1:** Hydrogen-bond geometry (Å, °)

*D*—H⋯*A*	*D*—H	H⋯*A*	*D*⋯*A*	*D*—H⋯*A*
N7—H8*C*⋯O5^i^	0.89	1.94	2.7169 (18)	145
N7—H9*A*⋯O1^ii^	0.89	2.12	2.8904 (18)	145
N7—H19*B*⋯O1^iii^	0.89	1.90	2.7657 (19)	164
N10—H1⋯O4^iv^	0.91	1.84	2.7367 (17)	168
O3—H3⋯O4^iv^	0.82	1.84	2.6415 (16)	164
O6—H6⋯O2^v^	0.82	1.80	2.5897 (16)	161

Symmetry codes: (i) 
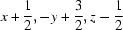
; (ii) 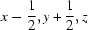; (iii) 
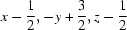
; (iv) 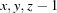; (v) 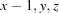.

**Fig. 1.**
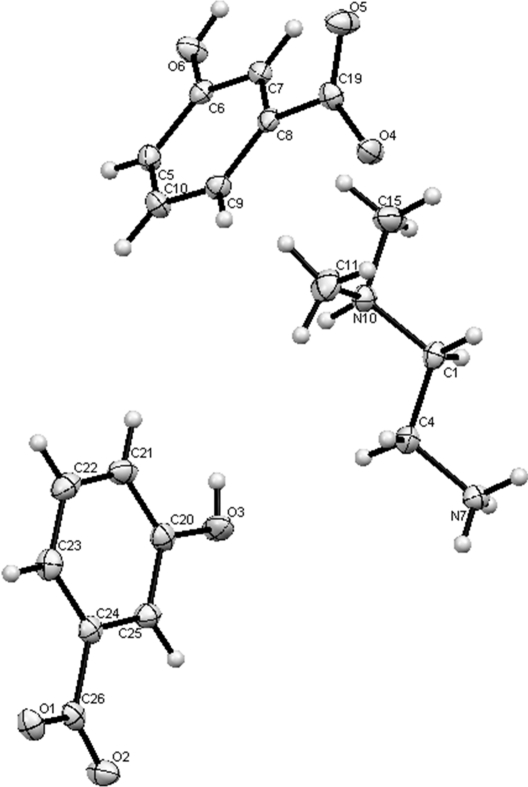
Molecular structure of N,N–dimethylethane-1,2-diaminium bis 3-hydroxybenzoate with atom labels and 50% probabilty ellipsoids.

**Fig. 2.**
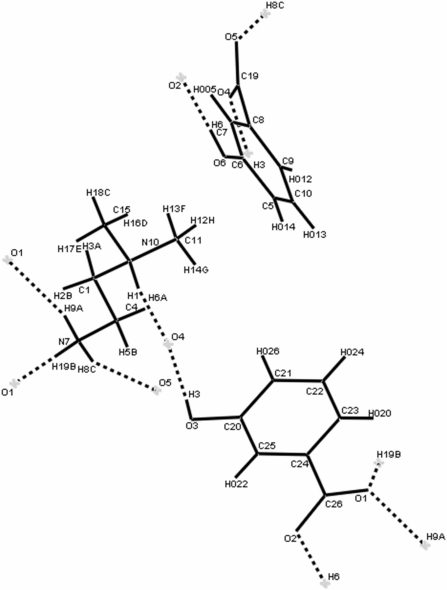
The H– bonded packing of N,N–dimethylethane-1,2-diaminium bis 3-hydroxybenzoate.

## supplementary crystallographic information

### Comment

*N*,*N*–Dimethylethane-1,2-diaminium bis 3-hydroxybenzoate [H_2_*DM*-en + 2(3HO-BA)] crystallizes in the centrosymmetric space group *Cc*. The molecular structure with the atom numbering scheme is shown in Figure 1. The all N–H bonds in H_2_*DM*-en (they are on average close to 0.9 Å) are shorter by about 0.1 Å when compared with those in other fully protonated polyamines (Thorn *et al.*, 2005; Doran *et al.*, 2003) but comparable with (Zhang *et al.*, 2007; Bujak *et al.*, 2002). At the same time, the three N—H bonds on N7 (primary N-atom) are equal in length (0.89 Å) and they are shorter by 0.02 Å than the N—H bond on tertiary N-atom (N10). In this crystal the polyammonium ion adopts an extended, all anti-conformation that minimizes electrostatic repulsions between protonated nitrogen atoms. There is a self-assembly pattern in the crystal through intermolecular noncovalent interactions (the hydrogen bonds of a type N—H···O and O—H···O). The O-atoms are known to participate as donors in intramolecular as well as intermolecular hydrogen-bonding interactions to provide various types of self-assembled networks. As an example, the O4-atom of the carboxylate group forms two intermolecular H-bonds, one with the diamine, N10—H1···O4, and another with hydroxy group of the benzoic acid, O3—H3···O4; both H-bonds are about 1.84 Å. The other O-atom of the carboxylic group is involved in only one intramolecular H-bond, namely N7—H19B···O5 involving terminal N-atom. In general, all O-atoms and H-atoms bonded to the N-atoms are involved in intramolecular interactions.

### Experimental

*DM*-en (0.83 ml, 8.97*M*) was mixed with 3-Hydroxy Benzoic acid (1 g m, 7.24 mmol) in water (1 ml). Colourless crystals obtained after 15 days of slow evaporation.

### Refinement

H atoms bonded to N and O atoms were located in a difference map and refined with distance restraints of O—H = 0.84 (2) and N—H = 0.87 (2) Å, and with *U*_iso_(H) = 1.2*U*_eq_(N,*O*). Other H atoms were positioned geometrically and refined using a riding model (including free rotation about the ethanol C—C bond), with C—H = 0.95–0.99 Å and with *U*_iso_(H) = 1.2 (1.5 for methyl groups) times *U*_eq_(C).

### Crystal data

**Table:**

C_4_H_14_N_2_^2+^·2C_7_H_5_O_3_^−^	*F*(000) = 776
*M**_r_* = 364.39	*D*_x_ = 1.352 Mg m^−^^3^*D*_m_ = 1.352 Mg m^−^^3^*D*_m_ measured by not measured
Monoclinic, *C**c*	Mo *K*α radiation, λ = 0.71073 Å
Hall symbol: C -2yc	Cell parameters from 4375 reflections
*a* = 14.5439 (3) Å	θ = 2.3–27.2°
*b* = 17.5881 (4) Å	µ = 0.10 mm^−^^1^
*c* = 7.7104 (2) Å	*T* = 293 K
β = 114.777 (1)°	Needle, colourless
*V* = 1790.76 (7) Å^3^	0.49 × 0.12 × 0.03 mm
*Z* = 4	

### Data collection

**Table:**

Bruker APEXII CCD diffractometer	4292 independent reflections
Radiation source: fine-focus sealed tube	3635 reflections with *I* > 2σ(*I*)
graphite	*R*_int_ = 0.048
φ and ω scans	θ_max_ = 28.0°, θ_min_ = 1.9°
Absorption correction: integration (*XPREP*; Bruker, 2005)	*h* = −19→19
*T*_min_ = 0.952, *T*_max_ = 0.997	*k* = −23→23
14114 measured reflections	*l* = −9→10

### Refinement

**Table:**

Refinement on *F*^2^	Secondary atom site location: difference Fourier map
Least-squares matrix: full	Hydrogen site location: inferred from neighbouring sites
*R*[*F*^2^ > 2σ(*F*^2^)] = 0.036	H-atom parameters constrained
*wR*(*F*^2^) = 0.077	*w* = 1/[σ^2^(*F*_o_^2^) + (0.037*P*)^2^] where *P* = (*F*_o_^2^ + 2*F*_c_^2^)/3
*S* = 0.96	(Δ/σ)_max_ < 0.001
4292 reflections	Δρ_max_ = 0.18 e Å^−^^3^
240 parameters	Δρ_min_ = −0.20 e Å^−^^3^
2 restraints	Absolute structure: Flack (1983), 2119 Friedel pairs
Primary atom site location: structure-invariant direct methods	Flack parameter: 0.00 (7)

### Special details

**Table:**

Geometry. All e.s.d.'s (except the e.s.d. in the dihedral angle between two l.s. planes) are estimated using the full covariance matrix. The cell e.s.d.'s are taken into account individually in the estimation of e.s.d.'s in distances, angles and torsion angles; correlations between e.s.d.'s in cell parameters are only used when they are defined by crystal symmetry. An approximate (isotropic) treatment of cell e.s.d.'s is used for estimating e.s.d.'s involving l.s. planes.
Refinement. Refinement of *F*^2^ against ALL reflections. The weighted *R*-factor *wR* and goodness of fit *S* are based on *F*^2^, conventional *R*-factors *R* are based on *F*, with *F* set to zero for negative *F*^2^. The threshold expression of *F*^2^ > σ(*F*^2^) is used only for calculating *R*-factors(gt) *etc*. and is not relevant to the choice of reflections for refinement. *R*-factors based on *F*^2^ are statistically about twice as large as those based on *F*, and *R*- factors based on ALL data will be even larger.

### Fractional atomic coordinates and isotropic or equivalent isotropic displacement parameters (Å^2^)

**Table:**

	*x*	*y*	*z*	*U*_iso_*/*U*_eq_	
O6	−0.04233 (9)	0.58513 (7)	0.31587 (16)	0.0236 (3)	
H6	−0.0877	0.6024	0.3410	0.035*	
O4	0.24002 (8)	0.70635 (7)	1.07125 (16)	0.0235 (3)	
N7	0.41688 (11)	0.92211 (8)	0.40647 (19)	0.0192 (3)	
H9A	0.4078	0.9518	0.4914	0.029*	
H19B	0.3910	0.9446	0.2926	0.029*	
H8C	0.4828	0.9142	0.4427	0.029*	
N10	0.19982 (10)	0.78889 (8)	0.3324 (2)	0.0202 (3)	
H1	0.2057	0.7653	0.2325	0.024*	
C7	0.05796 (12)	0.62591 (9)	0.6407 (2)	0.0174 (3)	
H005	0.0023	0.6493	0.6479	0.021*	
C1	0.25729 (12)	0.86195 (9)	0.3681 (2)	0.0189 (3)	
H3A	0.2578	0.8860	0.4817	0.023*	
H2B	0.2238	0.8961	0.2611	0.023*	
O5	0.08907 (9)	0.66203 (7)	1.02369 (18)	0.0284 (3)	
C6	0.04672 (12)	0.58805 (9)	0.4740 (2)	0.0177 (3)	
C8	0.15193 (12)	0.62880 (8)	0.7962 (2)	0.0170 (3)	
C4	0.36527 (12)	0.84847 (9)	0.3943 (3)	0.0225 (4)	
H6A	0.4014	0.8195	0.5102	0.027*	
H5B	0.3652	0.8193	0.2875	0.027*	
C19	0.16091 (12)	0.66816 (9)	0.9766 (2)	0.0186 (3)	
C9	0.23557 (12)	0.59331 (9)	0.7864 (2)	0.0190 (3)	
H012	0.2989	0.5961	0.8888	0.023*	
C10	0.22300 (13)	0.55372 (9)	0.6214 (2)	0.0215 (4)	
H013	0.2780	0.5287	0.6155	0.026*	
C5	0.12971 (12)	0.55123 (9)	0.4663 (2)	0.0206 (4)	
H014	0.1224	0.5249	0.3566	0.025*	
C15	0.09028 (13)	0.80408 (11)	0.2764 (3)	0.0297 (4)	
H18C	0.0823	0.8318	0.3766	0.044*	
H16D	0.0544	0.7567	0.2557	0.044*	
H17E	0.0636	0.8336	0.1610	0.044*	
C11	0.24122 (15)	0.73670 (10)	0.4997 (3)	0.0305 (4)	
H13F	0.2456	0.7630	0.6119	0.046*	
H14G	0.3075	0.7199	0.5180	0.046*	
H12H	0.1973	0.6935	0.4770	0.046*	
O1	0.80917 (9)	0.53105 (7)	0.53286 (16)	0.0231 (3)	
O2	0.78810 (9)	0.62504 (7)	0.32384 (18)	0.0267 (3)	
O3	0.43039 (9)	0.70905 (7)	0.11670 (19)	0.0272 (3)	
H3	0.3697	0.7025	0.0857	0.041*	
C23	0.60027 (13)	0.52862 (10)	0.4403 (2)	0.0229 (4)	
H020	0.6388	0.4881	0.5111	0.027*	
C26	0.75606 (12)	0.57987 (9)	0.4128 (2)	0.0192 (3)	
C25	0.58608 (13)	0.64466 (10)	0.2662 (2)	0.0199 (4)	
H022	0.6158	0.6824	0.2222	0.024*	
C24	0.64459 (12)	0.58440 (9)	0.3717 (2)	0.0184 (3)	
C22	0.49872 (13)	0.53375 (10)	0.4027 (3)	0.0256 (4)	
H024	0.4694	0.4966	0.4493	0.031*	
C20	0.48344 (12)	0.64932 (9)	0.2253 (2)	0.0204 (4)	
C21	0.43992 (13)	0.59385 (10)	0.2960 (2)	0.0235 (4)	
H026	0.3718	0.5969	0.2719	0.028*	

### Atomic displacement parameters (Å^2^)

**Table:**

	*U*^11^	*U*^22^	*U*^33^	*U*^12^	*U*^13^	*U*^23^
O6	0.0184 (6)	0.0337 (7)	0.0185 (6)	−0.0004 (5)	0.0076 (5)	−0.0060 (5)
O4	0.0171 (6)	0.0275 (7)	0.0248 (7)	−0.0043 (5)	0.0078 (5)	−0.0107 (5)
N7	0.0191 (7)	0.0217 (7)	0.0169 (7)	−0.0024 (6)	0.0077 (6)	−0.0010 (5)
N10	0.0213 (7)	0.0201 (7)	0.0199 (7)	−0.0032 (6)	0.0094 (6)	−0.0044 (6)
C7	0.0177 (8)	0.0160 (8)	0.0207 (8)	0.0001 (6)	0.0103 (7)	−0.0004 (7)
C1	0.0221 (8)	0.0167 (8)	0.0165 (8)	−0.0013 (7)	0.0068 (7)	−0.0007 (7)
O5	0.0234 (6)	0.0402 (8)	0.0262 (7)	−0.0092 (6)	0.0149 (6)	−0.0120 (6)
C6	0.0193 (8)	0.0163 (8)	0.0177 (8)	−0.0025 (6)	0.0081 (7)	0.0016 (6)
C8	0.0204 (8)	0.0130 (8)	0.0193 (8)	−0.0026 (6)	0.0099 (7)	0.0002 (6)
C4	0.0220 (9)	0.0171 (9)	0.0286 (10)	−0.0005 (7)	0.0107 (8)	−0.0008 (7)
C19	0.0180 (8)	0.0188 (8)	0.0168 (8)	0.0033 (7)	0.0051 (7)	0.0010 (7)
C9	0.0161 (8)	0.0180 (8)	0.0199 (8)	−0.0001 (6)	0.0047 (7)	0.0008 (7)
C10	0.0202 (8)	0.0196 (8)	0.0262 (10)	0.0041 (7)	0.0112 (7)	0.0001 (7)
C5	0.0248 (9)	0.0166 (8)	0.0224 (9)	−0.0012 (7)	0.0118 (8)	−0.0039 (7)
C15	0.0216 (9)	0.0359 (11)	0.0332 (11)	−0.0053 (8)	0.0132 (9)	−0.0075 (9)
C11	0.0375 (11)	0.0220 (9)	0.0344 (11)	−0.0023 (8)	0.0173 (10)	0.0045 (8)
O1	0.0219 (6)	0.0241 (6)	0.0191 (6)	0.0058 (5)	0.0046 (5)	0.0003 (5)
O2	0.0213 (6)	0.0294 (7)	0.0322 (7)	0.0044 (5)	0.0139 (6)	0.0091 (6)
O3	0.0149 (6)	0.0242 (6)	0.0393 (8)	0.0024 (5)	0.0081 (6)	0.0063 (6)
C23	0.0261 (9)	0.0210 (9)	0.0197 (9)	0.0017 (7)	0.0078 (7)	0.0026 (7)
C26	0.0190 (8)	0.0213 (9)	0.0151 (8)	0.0007 (7)	0.0050 (7)	−0.0051 (7)
C25	0.0202 (8)	0.0216 (8)	0.0198 (9)	−0.0031 (7)	0.0103 (7)	−0.0013 (7)
C24	0.0197 (8)	0.0196 (8)	0.0143 (8)	−0.0009 (6)	0.0057 (7)	−0.0034 (6)
C22	0.0271 (9)	0.0246 (9)	0.0271 (10)	−0.0066 (8)	0.0134 (8)	0.0003 (8)
C20	0.0194 (8)	0.0203 (9)	0.0187 (9)	0.0000 (7)	0.0053 (7)	−0.0034 (7)
C21	0.0174 (8)	0.0286 (9)	0.0240 (10)	−0.0042 (7)	0.0083 (7)	−0.0043 (7)

### Geometric parameters (Å, °)

**Table:**

O6—C6	1.358 (2)	C10—C5	1.383 (2)
O6—H6	0.8200	C10—H013	0.9300
O4—C19	1.2664 (19)	C5—H014	0.9300
N7—C4	1.480 (2)	C15—H18C	0.9600
N7—H9A	0.8900	C15—H16D	0.9600
N7—H19B	0.8900	C15—H17E	0.9600
N7—H8C	0.8900	C11—H13F	0.9600
N10—C11	1.489 (2)	C11—H14G	0.9600
N10—C15	1.490 (2)	C11—H12H	0.9600
N10—C1	1.494 (2)	O1—C26	1.262 (2)
N10—H1	0.9100	O2—C26	1.260 (2)
C7—C8	1.391 (2)	O3—C20	1.363 (2)
C7—C6	1.395 (2)	O3—H3	0.8200
C7—H005	0.9300	C23—C22	1.384 (2)
C1—C4	1.516 (2)	C23—C24	1.394 (2)
C1—H3A	0.9700	C23—H020	0.9300
C1—H2B	0.9700	C26—C24	1.518 (2)
O5—C19	1.246 (2)	C25—C24	1.389 (2)
C6—C5	1.393 (2)	C25—C20	1.393 (2)
C8—C9	1.397 (2)	C25—H022	0.9300
C8—C19	1.510 (2)	C22—C21	1.391 (2)
C4—H6A	0.9700	C22—H024	0.9300
C4—H5B	0.9700	C20—C21	1.392 (2)
C9—C10	1.393 (2)	C21—H026	0.9300
C9—H012	0.9300		
			
C6—O6—H6	109.5	C5—C10—C9	120.73 (15)
C4—N7—H9A	109.5	C5—C10—H013	119.6
C4—N7—H19B	109.5	C9—C10—H013	119.6
H9A—N7—H19B	109.5	C10—C5—C6	120.13 (15)
C4—N7—H8C	109.5	C10—C5—H014	119.9
H9A—N7—H8C	109.5	C6—C5—H014	119.9
H19B—N7—H8C	109.5	N10—C15—H18C	109.5
C11—N10—C15	110.88 (14)	N10—C15—H16D	109.5
C11—N10—C1	112.24 (13)	H18C—C15—H16D	109.5
C15—N10—C1	110.30 (13)	N10—C15—H17E	109.5
C11—N10—H1	107.7	H18C—C15—H17E	109.5
C15—N10—H1	107.7	H16D—C15—H17E	109.5
C1—N10—H1	107.7	N10—C11—H13F	109.5
C8—C7—C6	120.34 (15)	N10—C11—H14G	109.5
C8—C7—H005	119.8	H13F—C11—H14G	109.5
C6—C7—H005	119.8	N10—C11—H12H	109.5
N10—C1—C4	111.00 (13)	H13F—C11—H12H	109.5
N10—C1—H3A	109.4	H14G—C11—H12H	109.5
C4—C1—H3A	109.4	C20—O3—H3	109.5
N10—C1—H2B	109.4	C22—C23—C24	119.82 (17)
C4—C1—H2B	109.4	C22—C23—H020	120.1
H3A—C1—H2B	108.0	C24—C23—H020	120.1
O6—C6—C5	117.48 (15)	O2—C26—O1	125.20 (15)
O6—C6—C7	123.03 (14)	O2—C26—C24	117.38 (15)
C5—C6—C7	119.49 (15)	O1—C26—C24	117.43 (15)
C7—C8—C9	120.00 (15)	C24—C25—C20	120.85 (16)
C7—C8—C19	119.04 (14)	C24—C25—H022	119.6
C9—C8—C19	120.92 (15)	C20—C25—H022	119.6
N7—C4—C1	109.95 (13)	C25—C24—C23	119.48 (15)
N7—C4—H6A	109.7	C25—C24—C26	120.04 (15)
C1—C4—H6A	109.7	C23—C24—C26	120.47 (15)
N7—C4—H5B	109.7	C23—C22—C21	120.72 (16)
C1—C4—H5B	109.7	C23—C22—H024	119.6
H6A—C4—H5B	108.2	C21—C22—H024	119.6
O5—C19—O4	123.25 (15)	O3—C20—C21	123.19 (15)
O5—C19—C8	118.00 (14)	O3—C20—C25	117.46 (15)
O4—C19—C8	118.74 (14)	C21—C20—C25	119.35 (16)
C10—C9—C8	119.26 (16)	C22—C21—C20	119.76 (16)
C10—C9—H012	120.4	C22—C21—H026	120.1
C8—C9—H012	120.4	C20—C21—H026	120.1
			
C11—N10—C1—C4	64.80 (17)	C7—C6—C5—C10	−1.4 (2)
C15—N10—C1—C4	−171.02 (14)	C20—C25—C24—C23	−1.1 (2)
C8—C7—C6—O6	−178.08 (14)	C20—C25—C24—C26	179.29 (15)
C8—C7—C6—C5	1.8 (2)	C22—C23—C24—C25	0.0 (3)
C6—C7—C8—C9	−0.4 (2)	C22—C23—C24—C26	179.60 (15)
C6—C7—C8—C19	−178.15 (13)	O2—C26—C24—C25	−10.9 (2)
N10—C1—C4—N7	173.31 (13)	O1—C26—C24—C25	169.32 (15)
C7—C8—C19—O5	36.5 (2)	O2—C26—C24—C23	169.52 (15)
C9—C8—C19—O5	−141.30 (16)	O1—C26—C24—C23	−10.3 (2)
C7—C8—C19—O4	−142.72 (15)	C24—C23—C22—C21	0.4 (3)
C9—C8—C19—O4	39.5 (2)	C24—C25—C20—O3	−178.29 (15)
C7—C8—C9—C10	−1.5 (2)	C24—C25—C20—C21	1.8 (2)
C19—C8—C9—C10	176.23 (14)	C23—C22—C21—C20	0.3 (3)
C8—C9—C10—C5	1.9 (2)	O3—C20—C21—C22	178.71 (16)
C9—C10—C5—C6	−0.4 (3)	C25—C20—C21—C22	−1.3 (3)
O6—C6—C5—C10	178.49 (15)		

### Hydrogen-bond geometry (Å, °)

**Table:**

*D*—H···*A*	*D*—H	H···*A*	*D*···*A*	*D*—H···*A*
N7—H8C···O5^i^	0.89	1.94	2.7169 (18)	145
N7—H9A···O1^ii^	0.89	2.12	2.8904 (18)	145
N7—H19B···O1^iii^	0.89	1.90	2.7657 (19)	164
N10—H1···O4^iv^	0.91	1.84	2.7367 (17)	168
O3—H3···O4^iv^	0.82	1.84	2.6415 (16)	164.
O6—H6···O2^v^	0.82	1.80	2.5897 (16)	161.

Symmetry codes: (i) *x*+1/2, −*y*+3/2, *z*−1/2; (ii) *x*−1/2, *y*+1/2, *z*; (iii) *x*−1/2, −*y*+3/2, *z*−1/2; (iv) *x*, *y*, *z*−1; (v) *x*−1, *y*, *z*.

## References

[bb1] Bruker (2005). *APEX2*, *SAINT* and *XPREP* Bruker AXS Inc., Madison, Wisconsin, USA.

[bb2] Bujak, M. & Angel, R. J. (2006). *J. Phys. Chem. B*, **110**, 10322–10331. 10.1021/jp060652v 16722735

[bb3] Bujak, M. & Zaleski, J. (2002). *Main Group Met. Chem.* **25**, 571–573.

[bb4] Doran, M. B., Norquist, A. J. & O’Hare, D. (2003). *Inorg. Chem.* **42**, 6989–6991. 10.1021/ic034540j 14577764

[bb5] Farrugia, L. J. (1997). *J. Appl. Cryst.* **30**, 565.

[bb6] Flack, H. D. (1983). *Acta Cryst.* A**39**, 876–881.

[bb7] Macrae, C. F., Edgington, P. R., McCabe, P., Pidcock, E., Shields, G. P., Taylor, R., Towler, M. & van de Streek, J. (2006). *J. Appl. Cryst.* **39**, 453–457.

[bb8] Sheldrick, G. M. (2008). *Acta Cryst.* A**64**, 112–122. 10.1107/S0108767307043930 18156677

[bb9] Spek, A. L. (2009). *Acta Cryst.* D**65**, 148–155. 10.1107/S090744490804362X PMC2631630 19171970

[bb10] Thorn, K. J., Narducci Sarjeant, A. & Norquist, A. J. (2005). *Acta Cryst.* E**61**, m1665–m1667.

[bb11] Zhang, M., Sheng, L. T., Huang, X. E., Fu, R. B., Wang, X., Hu, S. M., Xiang, S. C. & Wu, X. T. (2007). *Eur. J. Inorg. Chem.* pp. 1606–1612.

